# Announcing the Minderoo – Monaco Commission on Plastics and Human Health

**DOI:** 10.5334/aogh.3916

**Published:** 2022-08-25

**Authors:** Philip J. Landrigan, Hervé Raps, Christos Symeonides, Thomas Chiles, Maureen Cropper, Judith Enck, Mark E. Hahn, Richard Hixson, Pushpam Kumar, Adetoun Mustapha, Yongjoon Park, Margaret Spring, John Stegeman, Richard Thompson, Zhanyun Wang, Megan Wolff, Aroub Yousuf, Sarah Dunlop

**Affiliations:** 1 Global Observatory on Planetary Health, Boston College, Chestnut Hill, MA, USA; 2 Centre Scientifique de Monaco, Monaco, MC; 3 Minderoo Foundation, Perth, WA, Australia; 4 Vice Provost for Research, Boston College, Chestnut Hill, MA, USA; 5 Economics Department, University of Maryland, College Park, MD, USA; 6 Beyond Plastics, Bennington College, Bennington, VT, USA; 7 Woods Hole Oceanographic Institution, Woods Hole, MA, USA; 8 University of Exeter, Exeter, UK; 9 United Nations Environment Programme, Nairobi, Kenya; 10 Nigerian Institute of Medical Research, Lagos, Nigeria; 11 University of Massachusetts Amherst, US; 12 Monterey Bay Aquarium, Monterey, CA; 13 International Marine Litter Research Unit, University of Plymouth, Plymouth, UK; 14 Empa – Swiss Federal Laboratories for Materials Science and Technology, Technology and Society Laboratory, Gallen, Switzerland; 15 School of Biological Sciences, The University of Western Australia, Australia

**Keywords:** plastic pollution, human health, environmental health, ocean health, plastic life cycle, microplastics

Plastic is the signature material of our age. In the 75 years since large-scale production began in the aftermath of World War II, plastic has transformed our world, supported many of the most significant advances of modern civilization, and enabled breakthroughs in virtually every field of human endeavor. But plastic also poses great and growing dangers to human health and the environment, harms that fall disproportionately on the world’s poorest and most vulnerable populations. The extent and magnitude of these dangers are only beginning to be understood.

In June 2022, in recognition of plastic’s growing dangers to human and planetary health, the United Nations Environment Assembly adopted a resolution to ‘End Plastic Pollution’ (UNEA Resolution 5/14). In this resolution, nations from around the world agreed to work together over the next two years to negotiate the first ever legally binding international treaty on plastic. Negotiation of this treaty will involve the crafting of global obligations to measure and reduce plastics across plastic’s full lifecycle; developing national action plans and national and international cooperative measures; devising a financial mechanism to support implementation of the treaty; and knowledge-sharing mechanisms to strengthen the science-policy interface. To support this historic process, robust analyses of plastic’s health impacts and science-based solutions to protect human health are urgently needed.

To meet this challenge, undertake a comprehensive analysis of plastic’s health impacts across its life cycle, and develop forward-looking, science-based recommendations that will prevent plastic-related disease and save lives, we have formed the Minderoo – Monaco Commission on Plastics and Human Health. This interdisciplinary Commission is comprised of scientists, healthcare workers, and policy analysts from around the world. It will be coordinated by the Global Observatory on Planetary Health at Boston College.

**Plastic production.** More than 8,300 million metric tons (MMt) of virgin plastic have been produced since 1950, approximately 1 metric ton (Mt) for every person on the planet [1]. Annual production of new plastic has grown from under 2 MMt in 1950 to more than 400 MMt today and is on track to double by 2040 and triple by 2060 [2,65]. The greatest recent increases are in the manufacture of single-use, disposable plastics, especially plastic packaging and other low-cost products and textiles. Single-use plastic now accounts for about 40% of total production and is projected to grow to two thirds of plastic production by 2060 [2].

More than 98% of all plastic is made from fossil fuels, mainly oil and gas [3,4]. Recent acceleration in plastic manufacture is driven by the combination of declining need for oil and gas for fuel as the global economy transitions to green energy, and enormous expansion in oil and gas production [4]. It is now economically attractive to use oil and gas as plastic feedstocks and the industry is pivoting away from fuel production and investing heavily in plastics and petrochemicals [4,5].

**Environmental Damage – Visible and Invisible Plastic.** Many plastic products are used only once or for a few times and then discarded. In 2015, only 30% of all plastic ever produced was still in use [1]. Single- and short-term use coupled with low recycling rates – less than 10% in most countries [6] – and plastic’s durability have resulted in massive global accumulation of plastic waste. Plastic waste is toxic [7] and contains multiple chemicals – substances intentionally or unintentionally inserted into plastic – including those used to convey specific properties such as color, flexibility, strength, fire resistance and water repellency. These chemicals include neurotoxicants, endocrine disruptors and carcinogens. These toxic chemicals can be released into the environment and into the bodies of living organisms [8,9].

An estimated 19.4 Mt of plastic waste are released annually to the environment, a volume that is projected from current trends to double to 38.4 Mt by 2060 [3]. The result has been massive global accumulation of plastic waste. Plastic waste is ubiquitous and is found in cities, suburbs, farms, beaches, the Amazon rain forest, the ocean depths, the Himalayan glaciers, the Australian outback, and the circumpolar regions [8]. Plastic burned in municipal dumps and waste-to energy facilities creates additional pollution [9].

The ocean has been badly damaged by plastic [10,11,12]. An estimated 8–12 MMt of plastic waste enters the ocean each year [1]. Macroplastics – the bottles, barrels, packaging materials and fishing gear that litter beaches, collect in mid-ocean gyres, and kill marine animals – are the most visible component of ocean plastic pollution, but are only the tip of the problem [10]. Much marine plastic is comprised of micro- and nanoplastic particles, including those formed through the degradation of plastic waste. These microscopic particles contaminate the water column, coat the sea floor, and enter the food web [8,9,10,11,12]. Many microplastics appear able to resist environmental degradation and could persist in the ocean for centuries [47].

**Plastic and Climate Change.** Plastic is linked to climate change [4,13,14]. Plastic manufacture is energy-intensive, and by 2050, manufacture of plastic and petrochemicals is projected to account for almost half of all growth in oil demand [15,16]. Plastic accounts for an estimated 4–5% of all greenhouse gas emissions, and by 2050, emissions from plastic are projected to rise to approximately 15–19% of total carbon emissions [17]. This estimate is probably low given the large and poorly quantified volumes of methane, natural gas’ principal component, is lost in drilling, transport, and storage; methane is a potent driver of global warming, with a heat-trapping potential 85 times greater than carbon dioxide over a 20-year period [18].

**Planetary Boundaries.** The Stockholm Environment Institute has introduced the concept of planetary boundaries to define the “safe operating space for humanity” [19], the conditions necessary for human societies to survive and thrive. These conditions include a temperate climate, adequate freshwater, sufficient fertile soil, abundant biological diversity, and a stratospheric ozone layer that protects all life on earth against solar radiation. A team of international scientists led by the Stockholm Environment Institute has recently concluded that production and environmental dissemination of novel chemical entities, including plastics, are increasing so rapidly and uncontrollably that they have outstripped global capacity for assessment and monitoring, and pose an existential threat to the survival of modern civilizations. Risk is high that pollution by plastic and other chemicals could – like climate change and biodiversity loss – lead to catastrophic disruption of the earth’s operating systems [20,21].

**Plastic and Human Health.** Plastic endangers human health. It causes disease, disability and premature death at every stage of its life cycle – from extraction of the oil and gas that are plastic’s main feedstocks, to transport, manufacture, refining, consumption, recycling, combustion, and disposal into the environment [22,23]. All of the health consequences of plastic production fall disproportionately on vulnerable, low-income, minority populations and on people in low-income and middle-income countries on the Global South. Children are especially susceptible.

Extraction of oil and gas by hydraulic fracturing (“fracking”) causes contamination of ground and surface water, air pollution, radiation releases, ecosystem damage, and earthquakes [22]. These exposures are associated with preterm birth, low birthweight, congenital heart defects, and childhood leukemia [24,25].

Gas transmission via pipelines, trucks, rail and ship results in fires and explosions [22]. The compressor stations located at intervals along gas pipelines release toxic and carcinogenic chemical vapors such as benzene and formaldehyde into surrounding communities [22].

Plastic manufacture exposes workers and residents of fenceline communities to multiple toxic chemicals. These include vinyl chloride monomer, 1, 3-butadiene, benzene, formaldehyde, and styrene as well as hazardous plastic additives and processing aids such as lead, tributyltins, phthalates, bisphenols, brominated flame retardants, and many per- and polyfluoroalkyl substances (PFAS). The health consequences of these exposures include increased incidence of hematologic, liver and brain cancers in plastic and chemical workers [26,27], and cancer clusters, including childhood leukemia clusters, in nearby communities [28,29]. Environmental injustice is pervasive in communities adjacent to plastic and other chemical manufacturing plants [30].

Air pollution produced by the combustion of discarded plastic exposes communities to fine particulate matter (PM_2.5_) air pollution, as well as to airborne dioxin (a known human carcinogen) and to lead and mercury (both neurotoxicants). PM_2.5_ pollution from burning plastic causes cardiovascular disease, stroke, chronic obstructive lung disease, lung cancer and diabetes in adults [31,32] and stillbirths [33], premature births [33], asthma [34], neurodevelopmental disorders and IQ loss in children [35].

The microplastic particles and fibers formed during production and use of plastic products, and through environmental degradation of plastic waste pose further risks to health [8,9]. Humans are exposed to these chemical-laden particles and fibers through consumption of contaminated seafood, fruits, vegetables, inhalation of airborne microplastic fibers, and ingestion of microplastics in drinking water [36,37,38]. Microplastic particles are reported to have been detected in human lung, colon, placenta, gut and stool [39,40,41,42,43,44], and nanoplastics are reported to have been detected in human blood [45].

The health impacts of microplastics are only beginning to be elucidated. Industrial exposures to microplastic fibers in textile workers are linked to lung diseases [37]. Microplastic particles are reported to have been detected in cirrhotic liver tissue in liver transplant patients [46]. The microplastic load in feces is reported to be associated with inflammatory bowel disease status [49].

The toxic chemicals that are added to plastic and found in microplastic particles are routinely detected in the bodies of people of all ages – in blood, urine, seminal and follicular fluid, amniotic fluid, cord blood, mothers’ urine during pregnancy, and breast milk [48,49]. *The extent to which additives that leach out from microplastic particles contribute to chemical body burdens is not known*. Various of these chemicals are known to increase risk for miscarriage, decreased birthweight, reproductive birth defects, neurodevelopmental disorders, metabolic and endocrine diseases, obesity, hypertension and cardiovascular disease, respiratory and allergic disease, adult reproductive disorders, and cancers [19]. Many have never been tested for safety or toxicity [50]. Almost nothing is known about the health consequences of exposures to mixtures of these chemicals [51].

**The Need.** Until now, the continuing accumulation of plastic waste in the earth’s environment, the health effects of endless growth in plastic production, and the increase in everyday use have not been comprehensively examined. Plastic’s contribution to the global burden of disease across its life cycle has not been quantified. Its economic costs are largely externalized and are not counted. Much of the public dialogue about plastic pollution has been limited to discussions about beach litter and the harms caused by plastic waste to whales, fish, seabirds, and turtles and has begun only recently to turn towards health [52].

The medical and public health communities have not systematically considered plastic’s effects on health and research into health effects has been piecemeal and fragmented. Community-based epidemiologic studies have documented the dangers of fracking [24,25]. Occupational studies have detailed the impacts of chemical and plastic production on disease and premature death in workers [26,27]. Biomonitoring surveys have recorded population-wide exposures in some countries to plastic chemicals [48]. Many studies of microplastics have been conducted, but until recently most of them have appeared in oceanographic and environmental journals where they are seldom seen by physicians or public health professionals.

**The Plan.** We have formed the Minderoo – Monaco Commission on Plastics and Human Health to bridge this gap in knowledge and to break down the silos that have separated the medical and public health communities from oceanographers and environmental researchers. The Commission plans to enumerate, and where possible to quantify, the multiple hazards that plastic poses to human health from extraction of its fossil carbon feedstocks through its everyday use, to its leakage and disposal into the environment. We will underscore the many unknowns and uncertainties that surround current knowledge of plastic’s health effects and identify research needs [10,51]. We plan, where the data permit, to estimate the health-related economic costs of plastic. In areas where sufficient data on health effects are not yet available, we will construct a framework to support the development of future economic estimates [53]. We will examine the ethical and moral implications of the unending production, consumption, and environmental disposal of plastics [54,55].

**The Solution.** The good news, which the Commission will forcefully emphasize, is that plastic pollution can be prevented [17,56]. The dangers that plastic poses to human health across its lifespan are formidable, but they can be overcome. The best evidence that plastic manufacture and pollution can be curbed comes from the experience of the many countries that have controlled air pollution [56], reduced airborne lead pollution by removing lead from gasoline [57], cleaned up polluted harbors, bays and estuaries [10], and come together through the United Nations’ Montréal Protocol to prevent destruction of the stratospheric ozone layer by chlorofluorocarbon chemicals [58]. This progress has been made possible by coordinated, multi-year, science-based strategies based on laws, policies and technology, backed by enforcement, and encouraged by incentives [56]. These same tools can be used to control plastic manufacture and pollution. Courageous and visionary political leadership has been and will continue to be critical.

Interventions against pollution have proven highly cost-effective. Every dollar invested since 1970 in the prevention of air pollution in the United States has yielded an economic return of $30 by decreasing health expenditures for pollution-related disease and increasing the economic productivity of a healthier, longer-lived population [59]. Removal of lead from gasoline has enhanced cognitive function and increased economic productivity in children around the world returning billions of dollars to national economies [57,60]. Clean-ups of polluted bays and harbors have prevented waterborne disease, restored commercial fisheries, increased tourism, and enhanced the economic value of coastal lands [10].

**Conclusion.** The Minderoo – Monaco Commission on Plastics and Human Health will present a comprehensive analysis of the hazards that plastic poses to human health and well-being at every stage of its life cycle. It will offer science-based recommendations designed to prevent plastic-related disease, disability, and premature death and to contain plastic’s externalized economic costs. It will envision a future in which essential uses of plastic are preserved, but the trivial and wasteful consumption of single-use and short-lived plastics is no more. It will point the way to a more circular global economy in which indestructible, environmentally persistent, and toxic plastics are replaced by safer, more sustainable alternatives [61].

Through the work of this Commission, we will educate physicians and public health workers about the full range of plastic’s hazards to human health. We will both ask and encourage these trusted professionals to use their privileged position in society to educate government leaders and policy makers about plastic’s clear and present dangers and to demand sweeping changes in regulation and control of chemicals and plastics.

The work of this Commission will inform the work of international leaders as they strive to fulfill the urgent call of the United Nations Environment Assembly to end plastic pollution and its unsustainable environmental, social, economic, and health-related impacts by negotiating a legally binding Global Plastics Treaty [62,63].

Our ultimate goal is to protect the earth, our Common Home [64], and to preserve this beautiful blue planet for our children, our grandchildren and the generations yet to come.

The Commission’s findings and recommendations will be released in Monaco in March 2023 during Monaco Ocean Week. At the same time, we plan to publish the Commission’s full report in *Annals of Global Health*.
